# Detection of Species Substitution in the Meat Value Chain by High-Resolution Melting Analysis of Mitochondrial PCR Products

**DOI:** 10.3390/foods10123090

**Published:** 2021-12-13

**Authors:** Jane Kagure Njaramba, Lillian Wambua, Titus Mukiama, Nelson Onzere Amugune, Jandouwe Villinger

**Affiliations:** 1 International Centre of Insect Physiology and Ecology (*icipe*), Nairobi P.O. Box 30772-00100, Kenya; njanekagure@gmail.com (J.K.N.); jandouwe@icipe.org (J.V.); 2 Department of Biology, University of Nairobi, Nairobi P.O. Box 30197-00100, Kenya; tmukiama@gmail.com (T.M.); namugune@uonbi.ac.ke (N.O.A.); 3 Animal and Human Health Division, International Livestock Research Institute, Nairobi P.O Box 30709-00100, Kenya

**Keywords:** species substitution, meat value chain, high-resolution melting analysis, *16S* rRNA, cytochrome c oxidase subunit 1, cytochrome b

## Abstract

Substituting high commercial-value meats with similar cheaper or undesirable species is a common form of food fraud that raises ethical, religious, and dietary concerns. Measures to monitor meat substitution are being put in place in many developed countries. However, information about similar efforts in sub-Saharan Africa is sparse. We used PCR coupled with high-resolution melting (PCR-HRM) analysis targeting three mitochondrial genes—cytochrome oxidase 1 (*CO1*), cytochrome b (*cyt b*), and *16S* rRNA—to detect species substitution in meat sold to consumers in Nairobi, Kenya. Out of 107 meat samples representing seven livestock animals, 11 (10.3%) had been substituted, with the highest rate being observed in samples sold as goat. Our results indicate that PCR-HRM analysis is a cost- and time-effective technique that can be employed to detect species substitution. The combined use of the three mitochondrial markers produced PCR-HRM profiles that successfully allowed for the consistent distinction of species in the analysis of raw, cooked, dried, and rotten meat samples, as well as of meat admixtures. We propose that this approach has broad applications in the protection of consumers against food fraud in the meat industry in low- and middle-income countries such as Kenya, as well as in developed countries.

## 1. Introduction

Food fraud, the intentional act of adulterating food products, often for dishonest economic gain, is an emerging concern in global trade as a crime against consumer rights and due to the inherent risks posed to public health. Food fraud is largely perpetrated by counterfeit descriptions of products with respect to their weights, details of origin, types of processing, and constituents (ingredients) [1]. Food fraud has been reported in most value chains, including spices [2,3], milk [4,5], edible oils [6], honey [7], fish [8,9], shellfish [10], cereals [11,12], vegetables [13], and meat [14,15], whereby the fraudulent substitution of ingredients or the adulteration of products with similar but cheaper options have been highlighted as major malpractice.

In the meat industry, the major fraudulent practice entails substituting meats of high commercial value with those from cheaper or undesirable species [14,16]. Major global incidences of species substitution have been reported, such as the horsemeat scandal in the UK and Ireland, where beef was substituted with horse meat [17], and in China, where mutton was substituted with murine meat [18]. In Kenya, the substitution of beef and chevron with bushmeat [19], in addition to reports of species substitution [20], necessitates the further study of efficient methods for detecting this malpractice in meat value chains. Species substitution in meat products inhibits fair trade [21] and raises ethical and religious concerns in which species substitutes are considered offensive [14,22]. Undeclared meat species are also a health liability to those with allergies [17] and are associated with public health safety risks, such as those posed by foodborne or zoonotic diseases. Substituted species utilized are frequently acquired from unconventional sources, such as wildlife (bushmeat), which may be subjected to unhygienic handling and may not undergo quality checks like meat inspection [23].

The detection of adulteration in the meat value chain relies on analytical techniques, such as chromatography, mass spectrophotometry, imaging, and serology, to identify particular contaminants, proteins, and metabolites, and to validate authenticity [15,16,24]. However, for the analysis of species substitution, DNA-based techniques have been increasingly adopted due to the inherent limitations in specificity and sensitivity associated with the aforementioned techniques [24], leading to the recognition of “Food Forensics” as a tool to investigate food fraud [13]. The use of DNA to identify species on the basis of universal barcoding markers has been reliably tested [16,19]. Previous studies have demonstrated the potential application of DNA sequencing to identify adulteration in meat products based on the *CO1* and *cyt b* genes [25,26]. While useful, the need for elaborate and relatively expensive post-PCR procedures, such as DNA sequencing, severely limits their usefulness in the routine monitoring of meat fraud in Kenya and other low-resource settings. A more recent technique, PCR coupled with high-resolution melting (HRM) analysis, allows for discriminating DNA variants by detection of nucleotide sequence differences, such as single nucleotide polymorphisms (SNPs) and insertions and deletions (indels) based on their melting profiles, hence enabling the genotyping of species [27].

High-resolution melting analysis utilizes dyes that fluoresce when bound to double-stranded DNA to allow for the discrimination of DNA sources based on their genetic disparity, which is evaluated by analyzing their dissociation curves [27,28]. The distinct curves, which are dependent on amplicon GC-content and sequence composition and length, allow for the distinction of closely related species [27], making it a useful tool in food authentication [28]. These curves are generated by measuring the fluorescence of amplicons as the temperature is gradually increased at a rate of 0.01–0.2 °C per second. Past studies have shown that in food analysis, HRM is an accurate, cost- and time-effective method for the detection of adulterants or the distinction of close species in fish and shellfish [9,10], meat [29], dairy products [5], honey [7], and saffron [3]. PCR-HRM approaches using multiple markers can increase the precision of analysis when some species are difficult to differentiate by one marker alone and can be evaluated by a process of elimination [19]. Such an approach has been used to identify vertebrate species in insect blood meals [30] and bushmeat [19].

Nevertheless, the accuracy of results for DNA-based analyses fundamentally depends on obtaining quality DNA to identify the species origins of frozen, cooked, processed, rotting, or mixed meat products [15,16,29], and the effect of different physicochemical states of meat on PCR efficiency remains understudied. Therefore, we studied the utility of a previously validated PCR-HRM analysis assay targeting *CO1*, *cyt b*, and *16S* rRNA genes [19] to investigate species substitution in Nairobi, Kenya. We also aimed to test the effect of various food processing techniques on meat samples (e.g., fresh, dried, cooked, or rotten meat), different DNA extraction protocols, and admixtures of different species on identification by PCR-HRM analysis.

## 2. Materials and Methods

### 2.1. Meat Samples

We purchased 107 meat samples (whole pieces) in November 2018 from randomly selected stalls in Nairobi’s major meat wholesale market (Burma market) and butcheries in the surrounding districts of Eastleigh, Kariokor, Kaloleni, Mukuru Village, Mathare, Jerusalem, Jericho, Ngara, and Makongeni. Meat samples of species commonly bought by households were purchased, including 61 cattle, 30 goat, 3 camel, 9 pig, and 4 chicken samples. Each 250 g sample was packed separately and transported in cooler boxes with ice packs to the lab. Sub-samples (1 g) were carefully excised from the internal portion of each sample to obtain two replicates. Sterile blades and fresh gloves were used for each sample on a sterile surface. The replicates were then stored in 2 mL cryovials at −80 °C, pending DNA extraction. Reference meat samples (voucher specimen) from 24 vertebrates that were characterized and archived from a previous study [19] were used as positive controls (Table S1, Figure S1). Genomic DNA was DNA-extracted from the sub-samples using the ISOLATE II Genomic DNA Extraction Kit (Bioline, London, UK) following the manufacturer’s instructions.

### 2.2. Identification of Vertebrate Sources of Meat by PCR-HRM

To identify the vertebrate species, DNA extracts from the test samples and positive controls (reference samples) were analyzed by PCR-HRM of vertebrate mitochondrial *cyt b*, *CO1*, and *16S* rDNA, as previously described, and validated [19,30]. Briefly, 10 µL PCR reactions were set up, each comprising 1× HOT FIREPol^®^ EvaGreen^®^ HRM Mix no ROX (Solis BioDyne, Tartu, Estonia), 0.5 µM of both forward and reverse primers (Table 1), 20 ng of DNA template, and nuclease-free water. Each run included a negative control in which ddH_2_O was added in place of DNA template. The PCR-HRM analyses were carried out in a RotorGene Q thermocycler (Qiagen, Germany) as described by Ouso et al. (2020) [19]. Briefly, the cycling conditions involved an initial hold at 95 °C for 15 min, followed by 45 cycles of denaturation at 95 °C for 20 s, annealing for 20 s at 56 °C, and an extension step at 72 °C for 30 s. This was followed by the final extension step, an additional 5 min at 72 °C. The amplicons were then gradually melted from 75 °C to 95 °C in 0.1 °C increments while recording the fluorescence every two seconds. The melt rate and normalized HRM graphs were generated from the fluorescence data using Rotor-Gene Q Series software (Version 2.3.1 build 49). Meat-source species were distinguished by analyzing the melt rate (melting temperature (Tm) peaks) and normalizing the profiles of the test samples against those of the reference species (voucher specimen). For species with single Tm peaks, we also examined the Tm deviation from the control, with similar species expected to have Tm shifts of <1 °C.

### 2.3. Analysis of Various Physicochemical Treatments of Meat on PCR-HRM

To study the effect of various physicochemical conditions of meat samples on species identification by PCR-HRM analysis, we utilized sub-samples from our collection of positive controls (see Table S1). Sub-samples of goat (*Capra hircus*), sheep (*Ovis aries*), pig (*Sus scrofa domesticus),* chicken (*Gallus domesticus*), cattle (*Bos taurus*), and camel (*Camelus dromedarius*) were exposed to different treatments to simulate fresh, dried, cooked (microwaved), and rotting/decomposed meat. This was achieved by obtaining four replicates weighing 60 mg from each sub-sample and treating them as follows: the first replicate was used as the fresh meat with no treatment applied; the second replicate was dried in an oven at 65 °C for 2 h; the third replicate was heated in a microwave oven for 12 min to simulate cooking; the fourth replicate was left on the lab bench for 72 h to decompose. Genomic DNA was extracted from the replicates of all samples as described above in Section 2.1, followed by PCR-HRM of the *CO1* gene, *cyt b*, and *16S* rRNA genes, as described in Section 2.2.

### 2.4. Analysis of Effect of Different Extraction Protocols

To study the impact of different DNA extraction protocols on species identification by PCR-HRM, sub-samples were obtained from two cattle, four goats, one sheep, and two camels, as described in Section 2.3, and subjected to four extraction protocols. Each sub-sample was divided into four 50 mg replicates and the DNA was extracted as follows: The first replicate was extracted using the ISOLATE II Genomic DNA Kit, as described in Section 2.1, and the second using the DNeasy Blood and Tissue Kit protocol (Qiagen, Germany) according to the manufacturer’s guidelines. The third replicate was extracted using a lab-optimized protocol described by Kipanga et al. [36]. The fourth replicate was extracted using a modified version of the aforementioned protocol, in which proteinase K was omitted during the cell lysis step. The extracted DNA was then standardized to 10 ng/µL and analyzed using the PCR-HRM of *CO1*, *cyt b*, and *16S* rRNA, as described in Section 2.2. The melt profiles were then compared to check for any differences in melt temperature or profile due to the varying extraction protocols.

### 2.5. Analysis of Species Admixtures in Meat by PCR-HRM

We investigated whether PCR-HRM could be successfully used to identify mixed species in meat, which is a common adulteration in processed meat. The following mixtures were prepared from the reference samples: cattle + sheep; sheep + goat; cattle + goat; cattle + camel; chicken + pork; chicken + Nile perch. In each case, triplicates containing 50 mg of each of the two species in the combinations above were placed into separate tubes. The genomic DNA was then extracted from the individual triplicates using the ISOLATE II Genomic DNA Kit, as described in Section 2.1. This protocol was selected because it produced high-quality DNA with a standardized concentration of ~40 ng/µL. This was followed by PCR-HRM analysis of the three mitochondrial markers (*CO1*, *cyt b*, and *16S* rRNA), as previously described. DNA extracts from individual reference samples, that is, individual voucher specimens of cattle, sheep, goat, chicken, pork, camel, and Nile perch, were also analyzed alongside the mixed samples.

### 2.6. DNA Sequencing for Species Confirmation and Statistical Analysis

To confirm the vertebrate species in the meat samples, the DNA was amplified using primers that target a longer segment (750 bp), the barcoding region of the *CO1* gene, as described previously [19,37]. This involved performing conventional PCR in 15-µL reaction volumes, which included 1× HOT FIREPol^®^ Blend Master Mix (Solis BioDyne, Tartu, Estonia), 0.5 µM concentrations of both forward (5′-TCT CAA CCA ACC ACA ARG AYA TYG G-3′) and reverse (5′-TAG ACT TCT GGG TGG CCR AAR AAY CA-3′) primers, and 2 µL of DNA template. The cycling conditions were those described by Ouso et al. [19]. The resulting amplicons were cleaned using the ExoSAP-IT protocol (USB Corporation, Cleveland, OH) and sequenced at Macrogen Inc. (Amsterdam, the Netherlands). Sequences were analyzed using Geneious version 11.1.5 [38,39] and queried against the GenBank nr database (http://www.ncbi.nlm.nih.gov/ accessed 10 March 2019) using the Basic Local Alignment Search Tool [40] and the Barcode of Life Database (BOLD; http://www.boldsystems.org (accessed 10 March 2019) [41]. The statistical software NCSS 2020 (NCSS, Kaysville, UT, USA; https://www.ncss.com/ (accessed on 28 August 2020) was used to create box plots of the variance in the melting temperatures observed using different extraction conditions and physicochemical treatments.

## 3. Results

### 3.1. Vertebrate Sources of Meat Sold in Butcheries in Nairobi

**Figure 1:**
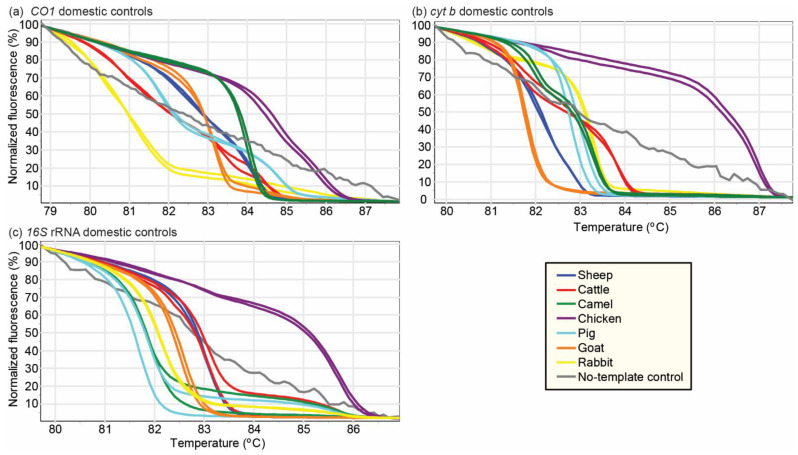
Distinct normalized PCR-HRM profiles of meat from seven reference livestock species. HRM profiles are represented as percent fluorescence with increasing temperatures for (**a**) *CO1*, (**b**) *cyt b*, and (**c**) *16S* rRNA markers. All unknown samples were compared against these reference profiles for identification of vertebrate species.

**Figure 2:**
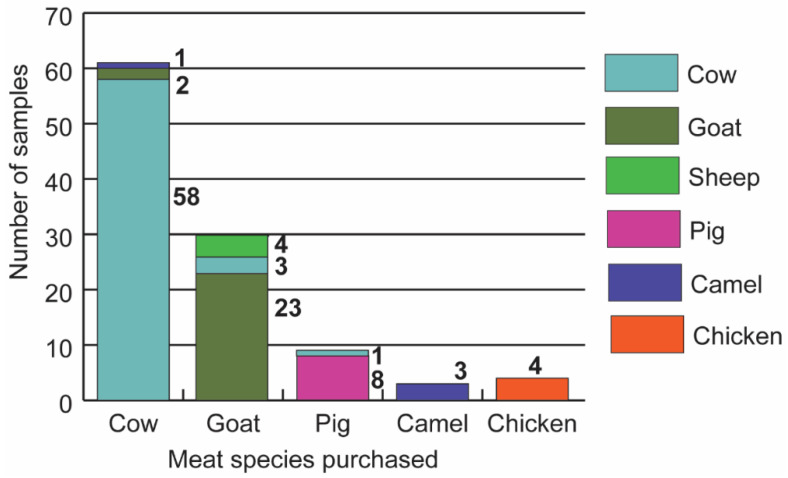
Species substitution of meat sampled in Nairobi. Stacked bar graph showing vertebrate species of meat identified by PCR-HRM against the identity of the species purchased in the meat market. Numbers against each species refer to *n*, the number of species identified using PCR-HRM. Species substitution was identified in cattle, goat, and pig samples.

**Figure 3:**
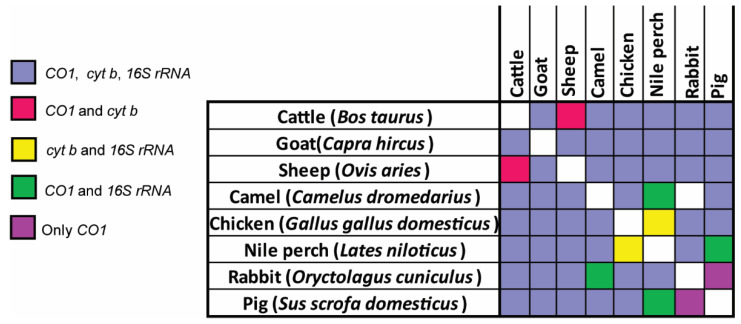
Pairwise discrimination of vertebrate sources of meat by PCR-HRM analysis: Three mitochondrial markers, *CO1*, *cyt b*, and *16S* rRNA, were compared. The abilities of these markers to distinguish eight different vertebrate species commonly consumed in Kenyan households were compared and the summary matrix was generated.

PCR-HRM analysis of the *cyt b*, *CO1*, and *16S* rRNA genes (Figure 1) of 107 meat samples revealed the vertebrate sources as 62 cattle (57.94%), 25 goats (23.36%), 8 pigs (7.47%), 4 camels (3.74%), 4 chickens (3.74%), and 4 sheep (3.74%). The identifications were confirmed by sequencing amplicons of the 750 bp segment of the *CO1* barcoding region. Sequence alignments of the PCR-HRM amplicons obtained from the reference vertebrate species are seen in Figure S1. Eleven (10.3%) meat samples were misidentified by sellers. Of 61 samples sold as beef, two were substituted with goat meat and one with camel meat. Of 30 samples sold as goat meat, four were mutton (sheep meat) and three were beef. One of the nine samples purchased as pork was beef (Figure 2). A pair-wise comparison of the amplicons allowed for the distinction of different species using the three primers (Figure 3).

### 3.2. Effect of Physicochemical Condition of Meat Samples on Vertebrate Species Identification by PCR-HRM

We found that the application of various treatments had minimal effect on the HRM melt profiles of the respective PCR amplicons. All samples, irrespective of the physicochemical condition, were amplified by at least one of the markers, and the vertebrate species could be reliably identified despite slight shifts in the melting temperature (Tm) of the resulting amplicons. Amplification was highest in the raw samples, with all of them being successfully detected using all three markers. However, we observed a relative reduction in the amplification of the markers from meat processed with different treatments. In the *CO1* gene, 8/16 of the microwaved, 2/16 of the rotten, and 4/16 of the oven-dried samples did not amplify, while in the assay targeting the *cyt b* marker, 10/16 of the microwaved samples, 2/16 of the oven-dried samples, and 1/16 rotten samples did not amplify. The *16S* rRNA did not amplify for 6/16 of the microwaved and 1/16 of the oven-dried samples.

**Figure 4:**
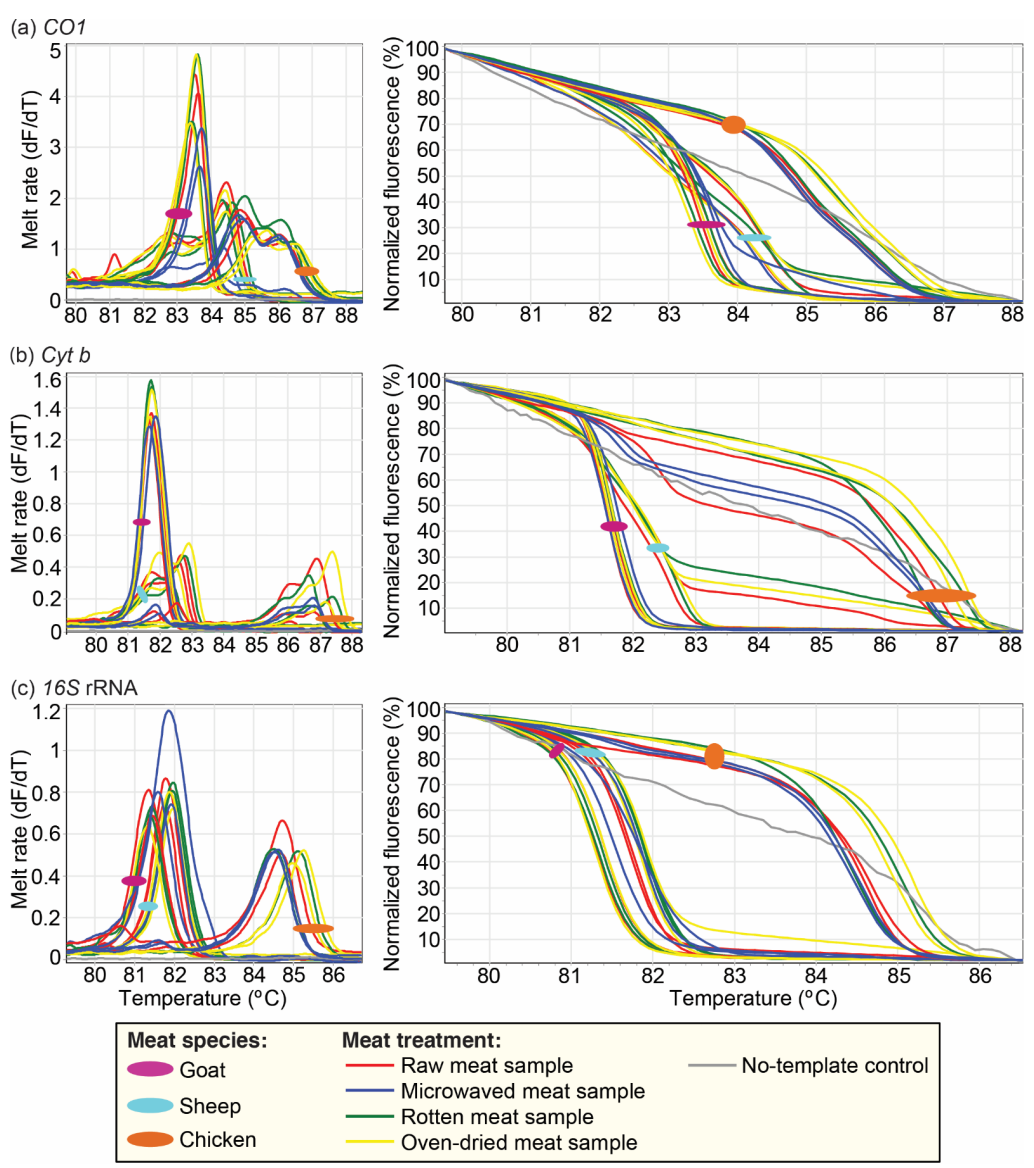
PCR-HRM profiles of representative reference samples exposed to different physicochemical conditions. Goat, sheep, and chicken meat samples were exposed in replicate to different conditions—raw, rotten, oven-dried, and microwaved. Their PCR-HRM profiles were then assessed using *CO1*, *cyt b*, and *16S* rRNA. For each marker, the HRM profiles are represented as melt rates and normalized HRM profiles. Melt rates are represented as changes in fluorescence units with increasing temperatures (dF/dT), and HRM profiles are represented as percent fluorescence with increasing temperatures for (**a**) *CO1*, (**b**) *cyt b*, and (**c**) *16S* rRNA markers.

Comparing the Tm of the PCR amplicons obtained from the oven-dried, cooked, and rotten meat to the raw samples indicated slight shifts. We observed the highest range in Tm shift with the *cyt b* marker when samples were exposed to the different conditions, whereas the Tm of the *CO1* marker was least affected by meat treatment. The shift in the Tm from the raw meat controls was <1 °C, with all markers for all samples. However, one microwaved cattle sample had a *16S* rRNA Tm shift of +1.63 °C. Based on the Tm of primary peaks, the widest range in Tm was seen in microwaved samples, followed by degraded samples, with oven-dried meat showing the least variation relative to the Tm of the corresponding raw meat (Figure 4, Figure S2). We further noted that the amplification of the *16S* rRNA marker resulted in single peaks in all the species tested, whereas the *cyt b* and *CO1* markers resulted in prominent secondary peaks that helped to distinguish cattle, sheep, and chicken (Figure S3). The camel samples had double peaks only in the *cyt b* region, whereas pig only had multiple peaks in the *CO1* region.

### 3.3. Effect of Different DNA Extraction Protocols on PCR-HRM

**Figure 5:**
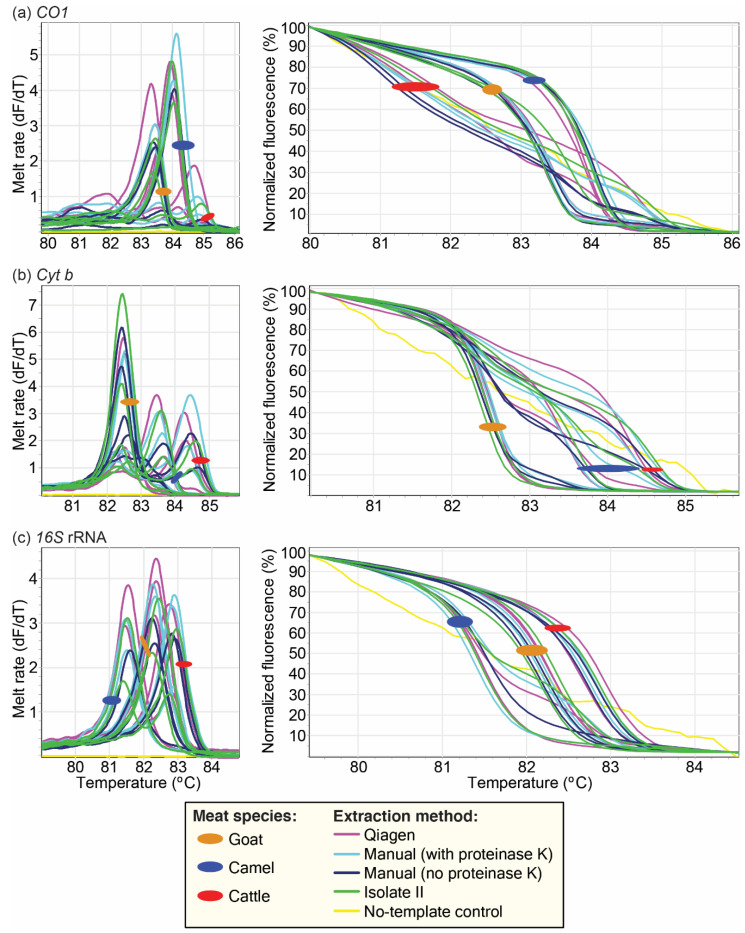
PCR-HRM profiles of representative reference samples whose DNA was extracted using different extraction protocols. DNA was extracted from goat, camel, and cattle meat samples using four different extraction protocols. These protocols included two kits, the DNeasy Blood and Tissue Kit protocol and the ISOLATE II Genomic DNA Extraction Kit, and two manual extraction protocols. Their PCR-HRM profiles were then assessed using *CO1*, *cyt b*, and *16S* rRNA. For each marker, the HRM profiles are represented as melt rates and normalized HRM profiles. Melt rates are represented as changes in fluorescence units with increasing temperatures (dF/dT) and HRM profiles are represented as percent fluorescence with increasing temperatures for (**a**) *CO1*, (**b**) *cyt b*, and (**c**) *16S* rRNA markers.

Using the different DNA extraction protocols, we observed similar melt profiles with minimal Tm shifts (<1 °C). The *CO1* marker had the widest range in Tm, followed by *cyt b* (Figure 5, Figure S4). The use of different extraction protocols did not result in overlapping of profiles of any of the species analyzed. All markers could be used to distinguish the species of the samples regardless of the extraction protocol used, with the exception of cattle and sheep, which yielded similar HRM melt profiles with the *16S* rRNA marker, as previously described [19].

### 3.4. Distinction of Species in Mixed Meat Samples Using PCR-HRM

**Figure 6:**
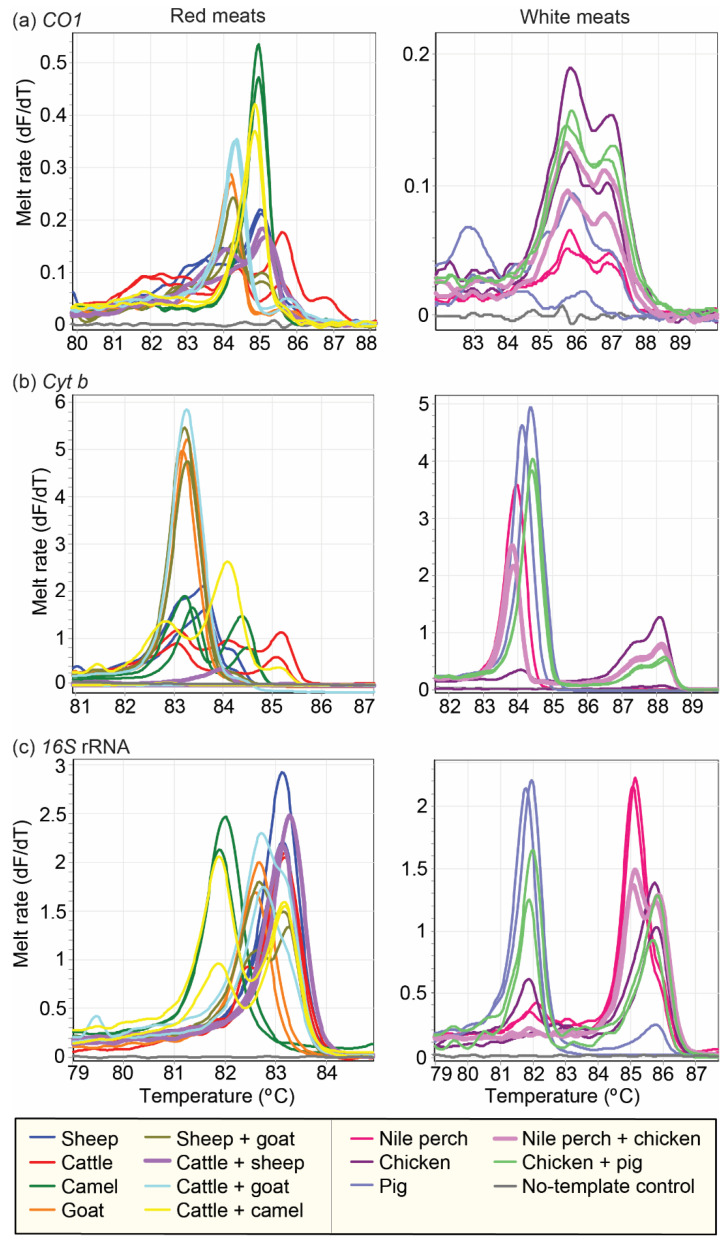
PCR-HRM melt rate profiles of pure and mixed meat samples assessed using three mitochondrial markers. The left column represents red meat sources (sheep, goat, cattle, and camel) and their corresponding mixtures, whereas the right column represents DNA from white meat sources (Nile perch, chicken, and pig) and their mixtures. Distinct melt rates are represented as changes in fluorescence units with increasing temperatures (dF/dT) for (**a**) *CO1*, (**b**) *cyt b*, and (**c**) *16S* rRNA markers.

Amplification targeting the marker *16S* rRNA gave the best resolution in distinguishing the individual vertebrate species in mixed meat samples (meat samples with two or more vertebrate species). The only mixed samples that could not be determined using the *16S* rRNA marker were mixtures of cattle and sheep meat, which could, however, be distinguished by the *cyt b* marker (Figure 3 and Figure 6). The *cyt b* marker clearly resolved white meat mixtures, including chicken and pork and Nile perch and pork, with individual HRM curves corresponding with the composite vertebrate species. However, differentiating sources of red meat using the *cyt b* marker was limited. For instance, all mixtures that contained goat meat only showed the *cyt b* melt profile of goat, and the “camel+cattle” admixture most resembled the camel sample, with a smaller third peak similar to the “cattle” samples. The melting profiles obtained from the *CO1* marker showed slight variations between the pure samples (meat samples with one vertebrate species) and the mixed meat samples.

## 4. Discussion

This study revealed significant levels of species substitution in products retailed in Nairobi’s major meat market. Goat meat had the highest levels of substitution, with mutton and beef being used as alternatives. Our results are comparable with those of Cawthorn et al. [15], who reported detecting mutton and beef as common substitutes in the meat value chain in South Africa. In Kenya, this substitution is likely to be driven by the relatively higher price of goat meat (USD 5.5–6.0 per kg) relative to mutton and beef (USD 3–4.5 per kg) [42]. Goat meat is preferred for preparing “Nyama Choma”, a roasted meat delicacy in eastern Africa that is increasingly being consumed in high quantities [43]. Growing evidence that associates the consumption of beef and mutton with a severe allergic reaction, termed “midnight anaphylaxis”, implies that substitution with these meats may pose a health risk to susceptible populations [44]. While the detection of these undeclared species could be a result of unintentional cross-contamination, such as from dirty knives or surfaces, we accounted for these potential mishaps by aseptically excising and testing inner parts of the raw meat samples. These results highlight the need for intensified surveillance of species substitution in the meat value chain in Kenya. Targeted surveillance may also be applied to goat value chains and other high-value species, in which adulteration could be linked to fraudulent financial gain.

This study demonstrates the utility of PCR-HRM for detecting multiple vertebrate species in meat products. We were able to detect composite vertebrate species in mixed meat samples by using different combinations of *CO1*, *cyt b*, and *16S* rRNA markers. The adulteration of meat with products from multiple species is increasingly being reported [17,45,46,47,48], thereby raising the demand for affordable and faster techniques for their detection. Many studies describing multi-species analyses of vertebrates in meat have utilized multiplex PCR [45,46,47,49]. While useful, multiplex PCR requires the use of expensive probes and post-PCR procedures, such as gel electrophoresis for the size separation of amplicons and/or DNA sequencing, thereby increasing analysis time, cost, and the risk of cross-contamination. Hence, PCR-HRM allows for real-time detection while minimizing downstream steps and costs. The PCR-HRM technique used here is hinged on measuring the dissociation rate of the PCR amplicons of three universal barcoding markers (*CO1*, *cyt b*, and *16S* rRNA) from their double-stranded to single-stranded forms when subjected to gradual heating. Therefore, this approach has wide application, as it is not limited to identifying specific species represented by species-specific primers. In a previous study, we demonstrated the utility of this PCR-HRM approach in distinguishing up to 32 vertebrate species [19], whereby DNA sequencing was performed only on representative samples for purposes of species confirmation, or to investigate samples with questionable or novel melt profiles.

This study shows that PCR-HRM would be particularly useful in investigating admixtures of vertebrate species in commercially processed meat products, such as sausages, kebabs, meatballs, and hams, which are frequently adulterated with multiple undeclared meats during processing [49]. In performing such analysis, our findings underpin the need to employ more than one marker to ensure accurate species identification in meat admixtures. Over-reliance on a single marker could result in overlaps in melt curves of different species, resulting in decreased sensitivity and poor resolution of meat mixtures [50]. Although the *16S* rRNA and *CO1* markers generated peaks that corresponded with the individual species that constituted the admixtures we tested, the *cyt b* marker showed a lower resolution, especially with red meats. This poor resolution could be attributed to the section of the *cyt b* region amplified, which resulted in melting profiles that were close to each other. Due to the unpredictability associated with meat mixtures, we hereby recommend the use of multiple mitochondrial markers, coupled with careful design and the selection of primers for PCR-HRM studies.

The application of various treatments of the meat samples allowed us to mimic the conditions and states of degradation that meat samples may be found in due to post-slaughter changes from cooking, sun-drying, or rotting [51]. The melting profiles were similar across raw, cooked, and rotten meat samples, with most samples having Tm shifts of <1 °C. However, exposure to heat treatment in the microwaved samples resulted in lower amplification rates and increased the *16S* rRNA marker Tm of one cattle sample by >1 °C. It has previously been demonstrated that the efficiency of PCR may be varied in meat samples subjected to different heat processes, possibly due to alterations in salt concentration and DNA fragmentation [52]. A shift of this magnitude was, however, not evident when targeting the *CO1* marker, showing that the use of this marker in conjunction with the others will improve species identification in meats exposed to heat post-slaughter. Our findings also imply that although reliable species distinction requires a Tm shift of <1 °C, it can be more in treated samples, hence the need for further studies to optimize acceptable thresholds. Nonetheless, across markers, species identification by PCR-HRM is reproducible despite varying physicochemical states of the meat samples.

Our results also demonstrate that the use of different DNA extraction protocols yielded only slight variations between same-species samples. Notably, the deviation from the control DNA isolation kit (the ISOLATE II commercial kit) was minimal (<1 °C), with the lab-optimized protocol having a slightly wider range in melting temperature compared to the other protocols used. The variation in melting temperature across different protocols was likely caused by the difference in salt concentrations of the DNA yielded. Cations such as Mg2+ and Na+ interact with the highly charged DNA polyanion, favoring DNA melting in conditions with lower Na+ concentrations [19,53]. Nevertheless, despite the marginal amplicon Tm shifts, melting profiles did not vary, confirming that PCR-HRM can be used with various DNA extraction protocols.

While PCR-HRM allows for the efficient and reliable differentiation of species substitution in the meat value chain, it is not without limitations. PCR-HRM relies on the identification of vertebrate species against reference samples that are run alongside the assays as positive controls. However, the samples selected as references may be subjective, determined by what researchers may deem as important, hence leaving out other species in the initial analysis. Nevertheless, our previous studies show that PCR-HRM analysis provides a degree of discovery of novel/uncharacterized species, which can be identified by DNA sequencing [19]. Furthermore, the mitochondrial markers *CO1* and *cyt b* are often used singly in species identification [17,46,50], but our results indicate the need to use multiple markers in tandem for accurate species identification. Finally, mitochondrial markers, which are commonly used for the DNA barcoding of species, are not appropriate for the quantification of species adulteration in meat mixtures because there are major differences in the gene copies of mtDNA markers in different species [54].

## 5. Conclusions

This study demonstrates the utility of PCR-HRM for the efficient and reliable detection of species substitution as a form of fraud in the meat value chain. We utilized this technique to identify species substitution in Nairobi’s major meat market. We also showed that PCR-HRM is a robust technique, providing reproducible results in both single and mixed-species samples despite variations in physicochemical properties or the presence of multiple DNA extraction protocols. Our study shows that PCR-HRM enables the identification of vertebrate species in meat samples without having to perform extensive DNA sequencing on most of the samples, making it the molecular tool of choice for the surveillance of food fraud in low-resource settings, such as those found in sub-Saharan Africa and other developing economies. Finally, this work demonstrates the importance of using multiple mitochondrial markers, including *CO1*, *cyt b*, and *16S* rRNA, to accurately distinguish species.

## Figures and Tables

**Table 1:** Oligonucleotide primers used for vertebrate identification.

Target Gene	Primer Name	Primer Sequence (5′-3′)	Amplicon Size (bp)	Citation
*CO1*	Uni-Minibar-F1	TCCACTAATCACAARGATATTGGTAC	205	[19,31,32]
	Ronping_R	TATCAGGGGCTCCGATTAT		
*16S* rRNA	Vert*16S* For	GAGAAGACCCTRTGGARCTT	200	[33]
	Vert*16S* Rev	CGCTGTTATCCCTAGGGTA		
*cyt b*	Cyt b For	CCCCTCAGAATGATATTTGTCCTCA	383	[34,35]
	Cyt b Rev	CATCCAACATCTCAGCATGATGAAA		

## Data Availability

The data contained in this study are available in the main manuscript, and in the supplementary materials.

## Supplementary Materials

The following are available online at https://www.mdpi.com/article/10.3390/foods10123090/s1, Table S1: Metadata of reference species. Metadata of voucher specimens used as positive controls in this study. Figure S1: Sequence alignments of reference species (A) *CO1*, (B) *cyt b*, and (C) *16S* rRNA PCR products used to differentiate livestock species by HRM analysis, Figure S2: Box plots of peak PCR-HRM melting temperatures of DNA from meat samples exposed to different physicochemical conditions, Figure S3: PCR-HRM distinction of double-peaked profiles, Figure S4: Box plots of peak PCR-HRM melting temperatures of DNA extracted from meat samples using different protocols.
