# Supplementary material for: Detection of Species Substitution in the Meat Value Chain by High-Resolution Melting Analysis of Mitochondrial PCR Products
Source: Foods. 2021 Dec 13;10(12):3090. doi: 10.3390/foods10123090 (PMC8702074; doi:10.3390/foods10123090)
Supplement: Supplementary file 1 [file foods-10-03090-s001.zip › foods-1419760-supplementary.pdf]

Supplementary Table S1: Metadata of voucher specimen. Metadata of voucher specimen used as positive controls in this study. Meat samples from known vertebrate species selected for use as positive controls in the PCR-HRM analyses.

Species	Scientific name	Origin of sample	GPS Address	Reference	No. of replicates
**Chicken**	*Gallus gallus domesticus*	*icipe*	1°13'13.8"S, 36°53'48.7"E	(Ouso et al., 2020)	3
**Goat**	*Capra hircus*	*icipe*	1°13'13.8"S, 36°53'48.7"E	(Ouso et al., 2020)	3
**Rabbit**	*Oryctolagus cuniculus*	*icipe* insectary	1°13'13.8"S, 36°53'48.7"E	-	3
**Cattle**	*Bos taurus*	*icipe*	1°13'13.8"S, 36°53'48.7"E	(Ouso et al., 2020)	3
**Pig**	*Sus scrofa domesticus*	*icipe*	1°13'13.8"S, 36°53'48.7"E	(Ouso et al., 2020)	3
**Camel**	*Camelus dromedarius*	*icipe*	1°13'13.8"S, 36°53'48.7"E	-	3
**Sheep**	*Ovis aries*	*icipe*	1°13'13.8"S, 36°53'48.7"E	(Ouso et al., 2020)	3
**Nile perch**	*Lates niloticus*	*icipe*	1°13'13.8"S, 36°53'48.7"E	(Ouso et al., 2020)	3

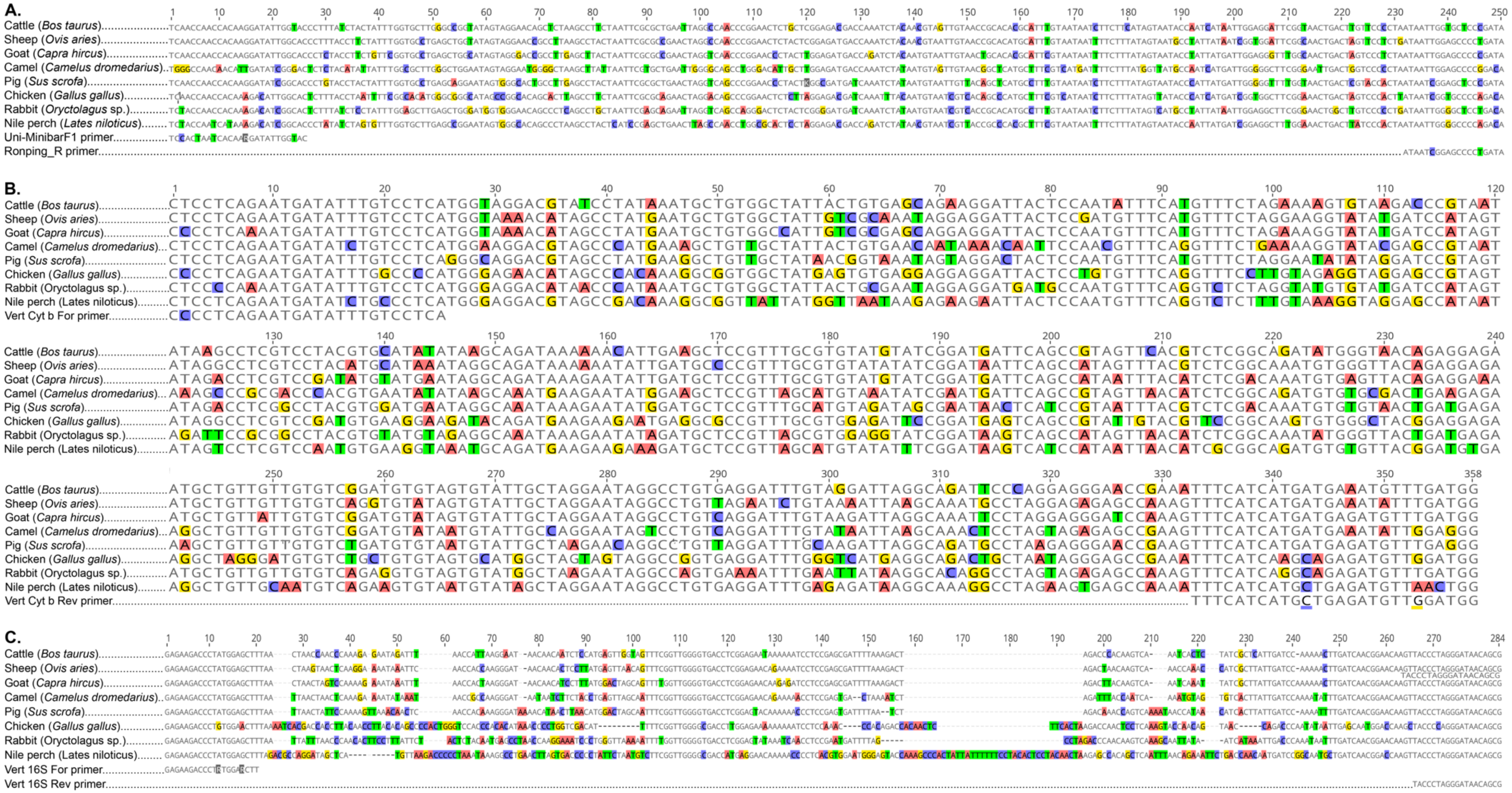

Supplementary Figure S1: Sequence alignments of voucher specimen (A) *COI*, (B) *cyt b*, and (C) *16S rRNA* PCR products used to differentiate livestock species by HRM analysis.

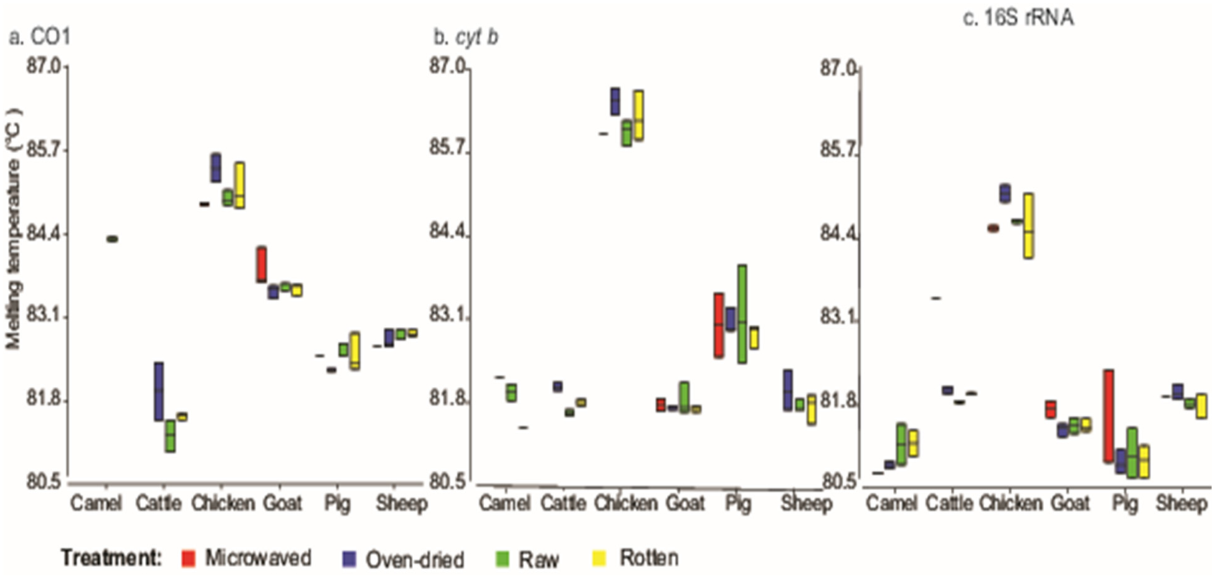

Supplementary Figure S2: Box plots of peak melting temperatures (°C) of meat samples exposed to different physicochemical conditions. Replicate meat samples from goat, sheep, pig, cattle, camel, and chicken were exposed to different conditions; raw, rotten, oven-dried, and microwaved. The peak PCR-HRM melt rate temperatures were plotted for a) *CO1*, b) *cyt b*, and c) *16S rRNA* markers.

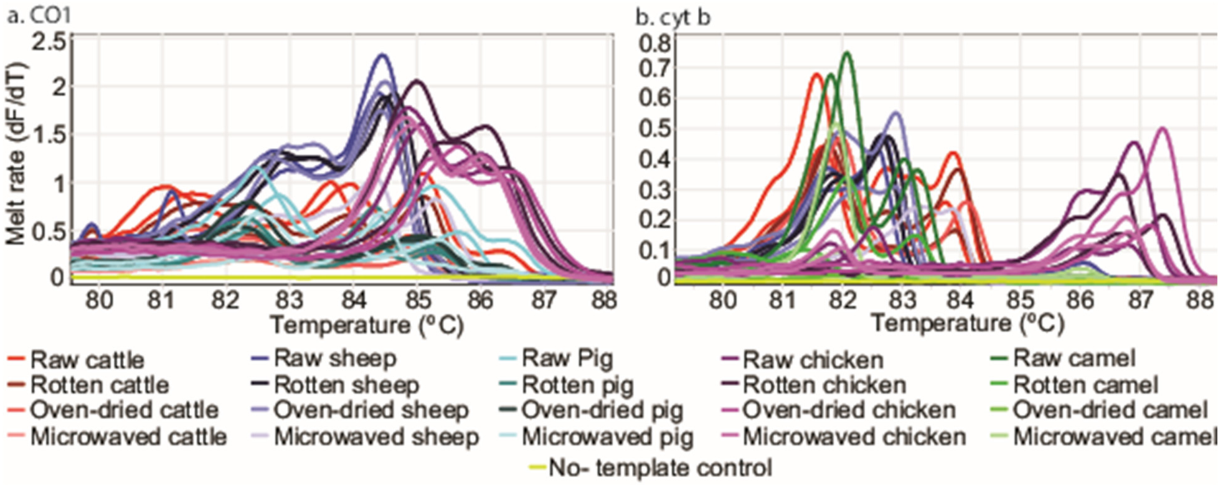

Supplementary Figure S3: PCR-HRM profiles showing the distinction of species presenting with double peaks. Unique double peaks produced by samples from cattle, sheep, pig, chicken and camel samples when targeting *cyt b* and *CO1* allowed for easier identification of these vertebrates. This differentiation relied on the melt profiles, through the analysis of multiple peaks produced in the amplification of a) *CO1* and b) *cyt b.*

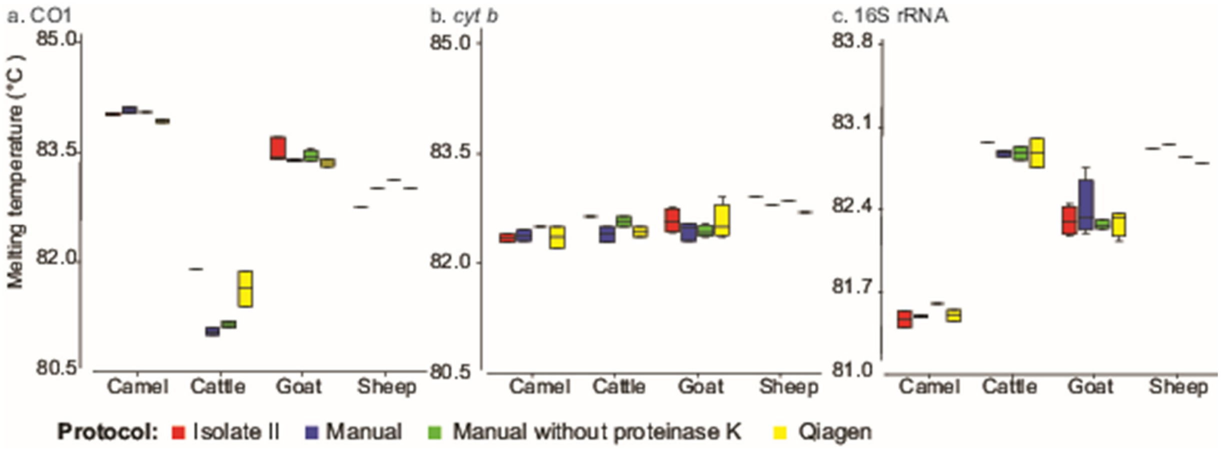

Supplementary Figure S4: Box plots of peak melting temperature (°C) seen in meat samples extracted using different extraction protocols. DNA from four vertebrate species (two cattle, four goats, one sheep and two camels) were extracted in replicate using two commercial kits; DNeasy Blood and Tissue Kit protocol, the ISOLATE II Genomic DNA Extraction Kit, and two manual extraction protocols. The peak PCR-HRM melt rate temperatures were plotted for a) *CO1*, b) *cyt b*, and c) *16S rRNA* markers.
